# Comment on Jankovic et al. Understanding the Benefits of CO_2_ Laser Treatment for Vulvovaginal Atrophy. *Medicina* 2024, *60*, 1059

**DOI:** 10.3390/medicina61050831

**Published:** 2025-04-30

**Authors:** Catherine Broomfield, Fiona Li, Jason Abbott

**Affiliations:** School of Clinical Medicine, University of New South Wales, Sydney 2052, Australia; fiona.li@unsw.edu.au (F.L.); j.abbott@unsw.edu.au (J.A.)

We write with concerns regarding the recent publication of “Understanding the Benefits of CO_2_ Laser Treatment for Vulvovaginal Atrophy” [1].

There are considerable similarities noted between this publication [1] and a second publication by the same group [2], with both addressing similar objectives, using similar outcome measures (VHIS and FSFI) and drawing similar conclusions based on the data. The only substantial difference that can be identified is that the current publication [1] has less of a focus on urinary/prolapse symptoms than the second publication [2]. 

In terms of the sample, we are concerned that there is a subset of the same participants used in the current publication (*n* = 84) [1] that has been used in the second publication (*n* = 73) [2]. The recruitment period of the second publication (June to November 2022) [2] falls within that of the current publication (January 2022 to March 2023) [1], yet there is no mention of a link between the two publications and a lack of transparency as to whether one of the publications is a secondary analysis of data already published from the same study. Given there is a risk that replicated samples across publications may be duplicated in systematic reviews and meta-analyses, it is imperative that any relationship that may exist between these two publications be made explicit to readers.

A final concern is the conclusions being drawn on the efficacy of CO_2_ laser therapy for women who have experienced VVA symptoms. What is noticeably missing in this publication [1] is the acknowledgement of the numerous RCTs on this topic that have found no significant differences in efficacy between CO_2_ laser therapy and sham control conditions [3,4,5]. This has been supported by a meta-analysis of RCTs investigating this topic [6]. The authors have correctly identified the limitations of their study, which includes the lack of a control group, no randomization of participants and the exclusion of long-term follow-up [1]—all of which are essential when determining the efficacy of CO_2_ laser treatment for the treatment of VVA-associated genital discomfort. To advance this area further, it is imperative that well-conducted randomized trials prevail over small-scale prospective analyses that are prone to bias.
